# The relationship between the functional status of upper extremity motor neurons and motor function and prognosis in stroke patients

**DOI:** 10.3389/fneur.2024.1466252

**Published:** 2024-09-20

**Authors:** Xiaoyi Li, Zhen Shao, Zhi Li, Xiupan Wei, Lijuan Zong, Pei Wang, Ting Zhou, Hongxing Wang

**Affiliations:** ^1^Department of Rehabilitation Medicine, Zhongda Hospital Southeast University, School of Medicine, Southeast University, Nanjing, China; ^2^Department of Rehabilitation Medicine, Affiliated Hospital of Xuzhou Medical University, Xuzhou, China

**Keywords:** stroke, motor neurons, repeater F-waves, MUNE, upper extremity motor function

## Abstract

**Introduction:**

This study investigates the correlation between neuroelectrop-hysiological assessments such as motor unit number estimation (MUNE) and F-waves with upper extremity motor function and one-year prognosis in stroke patients.

**Methods:**

Neuroelectrophysiological assessments of the abductor pollicis brevis muscle, including MUNE and F-waves, were conducted. Upper extremity motor function was evaluated using the Fugl-Meyer Assessment of Upper Extremity (FMA-UE) and the Modified Ashworth Scale (MAS). Pearson correlation and multiple linear regression analyses were performed to explore the relationship between upper extremity motor function and variables such as MUNE and F-waves. ROC curve analysis assessed the predictive ability of MUNE and F-waves for upper extremity motor function, and binary logistic regression analysis examined factors related to motor function improvement 1 year post-discharge.

**Results:**

A total of 130 patients were ultimately included. Significant differences in MUNE and occupancy rate of non-repeater F-waves (non-ORF) were found between hemiplegic and unaffected sides (*p* < 0.001), with a significant difference in F-wave mean latency (*p* < 0.05). Pearson correlation analysis showed a positive correlation between FMA-UE at admission and hemiplegic side’s MUNE and non-ORF (*p* < 0.001). Multiple linear regression indicated that hemiplegic side’s MUNE (*β* = 0.88, *p* < 0.001) and non-ORF (*β* = 0.275, *p* = 0.005) influenced FMA-UE. ROC analysis demonstrated higher predictive ability for hemiplegic side’s MUNE (AUC = 0.696, *p* < 0.001) than non-ORF (AUC = 0.622, *p* = 0.018). Binary logistic regression showed that hemiplegic side’s MUNE was associated with FMA-UE improvement 1 year post-discharge.

**Conclusion:**

MUNE and F-waves are correlated with upper extremity motor function in patients, reflecting their motor function status. These indicators have good predictive value for motor function and are associated with the prognosis of upper extremity motor function to a certain extent.

## 1 Introduction

Stroke is one of the leading causes of disability and death worldwide. Research indicates that approximately 80% of acute stroke patients experience upper extremity motor dysfunction, and 50–60% of these patients continue to suffer from this dysfunction 6 months post-stroke (1). This significantly impacts patients’ quality of life and daily living abilities, placing a heavy burden on patients, their families, and society (2). Evidence-based medicine has proven that rehabilitation plays a crucial role in optimizing functional recovery, with rehabilitation assessment being the foundation of treatment. Therefore, accurately and systematically quantifying upper extremity motor dysfunction in patients is a complex yet critical issue in rehabilitation (3).

Stroke primarily affects the upper motor neuron regions. However, studies have shown that post-stroke, patients often exhibit secondary lower motor neuron dysfunction (4, 5). This dysfunction is related to damage to the upper motor neurons (corticospinal tract) and the loss of trophic support to the target organs (skeletal muscles), with the extent of lower motor neuron damage potentially corresponding to the severity of corticospinal tract damage and muscle paralysis (6). Although changes in lower motor neuron function are crucial for post-stroke skeletal muscle and motor control capabilities, current stroke treatment and rehabilitation guidelines pay limited attention to lower motor neuron dysfunction, and clinical assessments rarely evaluate related indicators. Thus, accurately assessing lower motor neurons is vital for understanding the recovery of motor dysfunction post-stroke.

The commonly used physical examinations and functional scales in clinical practice focus on muscle function assessment but cannot accurately reflect lower motor neuron dysfunction. They are also subjective and prone to biases influenced by personal experience (7, 8). In contrast, neuroelectrophysiological assessment is an extension of the nervous system examination, capable of evaluating the integrity of lower motor neurons (9). As a non-invasive and repeatable method, it aids in developing rehabilitation treatment plans and provides quantitative reference data for functional evaluation (10). Among them, the abductor pollicis brevis, innervated by the median nerve, is frequently selected for nerve electrophysiological examination due to its high responsiveness to electrical stimulation and excellent reproducibility of results. Moreover, this muscle often exhibits pathological spontaneous activity during needle electromyography (EMG), a phenomenon commonly observed in the distal muscles of the hemiplegic upper limb and hand (11, 12).

In recent years, neuroelectrophysiological assessment such as motor unit number estimation (MUNE) and F-waves have been increasingly used for quantitative measurement of motor units and motor neuron functional status (13, 14). However, there are currently few studies on the relationship between motor neuron functional status and patients’ motor function and prognosis post-stroke. Therefore, this study aims to investigate the correlation between neuroelectrophysiological assessment (MUNE and F-waves) of the median nerve in the upper extremity of stroke patients and their motor function and one-year prognosis after discharge, exploring the predictive value of these indicators for patients’ upper extremity motor function and prognosis.

## 2 Methods

### 2.1 Subjects

From November 2020 to May 2023, 190 stroke patients admitted to the Rehabilitation Medicine Department of Zhongda Hospital Southeast University, were consecutively selected for this study. The inclusion criteria were as follows: (1) first-time diagnosis of cerebral infarction or cerebral hemorrhage, confirmed by cranial CT or MRI, with lesions confined to one cerebral hemisphere according to WHO standards; (2) unilateral limb motor dysfunction of varying degrees; (3) age between 16 and 80 years, with stable vital signs, clear consciousness, and no severe cognitive impairment; (4) disease duration of 0 to 6 months; (5) good cardiopulmonary function, with no swelling or skin damage in the upper extremities; and (6) voluntary participation with signed informed consent. The exclusion criteria included: (1) unstable condition or non-cooperation; (2) previous damage to the central nervous system other than stroke; (3) previous peripheral neuropathy, radiculopathy, neuromuscular junction disorders, or motor neuron diseases, and other diseases those may affect peripheral nerves or peripheral neuropathy as assessed by history, neurologic examination, and electrodiagnosis; (4) other conditions causing increased muscle tone or previous use of medications affecting muscle tone; (5) previous upper extremity motor dysfunction due to trauma or osteoarthropathy; and (6) participation in other clinical trials. Patients who were found to not meet the inclusion criteria after enrollment or had incomplete case data that precluded efficacy evaluation were excluded. Patients who could not continue participation or voluntarily withdrew during the evaluation process were considered dropouts. This study was approved by the Clinical Research Ethics Committee of Zhongda Hospital Southeast University (approval number: 2022ZDSYLL397-P01).

Study Procedures All eligible inpatients or their immediate family members were interviewed to obtain consent. General information, including age, gender, type of stroke, hemiplegic UE, right handed or left handed and disease duration, were collected and recorded. In this study, following rehabilitation assessment, individualized routine rehabilitation training programs were developed for patients based on their existing functional impairments. These programs included physical therapy, occupational therapy, acupuncture,and other treatments. The aforementioned treatments were administered one time daily, 6 days per week, for 3 weeks totally. After discharge, professional healthcare personnel followed up with patients through outpatient visits, telephone calls, or community visits 1 year post-discharge. The primary outcome measure was the Fugl-Meyer Assessment of Upper Extremity (FMA-UE) score 1 year after discharge.

### 2.2 Motor function assessment

#### 2.2.1 FMA-UE

The FMA-UE consists of 33 items, each scored up to 2 points. Except for the “presence of upper extremity reflex activity,” which is scored as 0 or 2 points, the remaining 31 items are scored as 0, 1, or 2 points, with a total possible score of 66. Higher scores indicate better upper extremity motor function. The difference between the first assessment (at hospital admission) and the final follow-up assessment (1 year after discharge) in the FMA-UE score was used to determine improvement in upper extremity motor function. In this study, the minimal clinically important difference (MCID) for the FMA-UE (defined as an FMA-UE change score = 6) was used as the threshold (15). Patients with an FMA-UE change score of 6 or more were classified as having improved function, while those with a score of less than 6 were classified as having unimproved function. The MCID was chosen as the binary classification threshold because it represents a meaningful clinical improvement for patients (16).

#### 2.2.2 MAS

The MAS was used to assess muscle tone in the hemiplegic side’s wrist flexors. Only changes in muscle tone during passive wrist extension in the hemiplegic upper extremity were evaluated. Patients were seated with their bodies upright, elbows flexed at 90°, and forearms in pronation. Muscle tone was graded as 0, 1, 1+, 2, 3 and 4, with higher scores indicating greater muscle tone.

### 2.3 Neuroelectrophysiological assessment

Neuroelectrophysiological assessment was conducted using a Haishen NDI-094 electromyography evoked potential instrument in a quiet room at a temperature of 22–25°C. The patient’s skin temperature was maintained above 32°C, and the local skin of the recording and stimulation sites was cleaned. The patient was placed in a supine or sitting position, with the muscles to be tested kept relaxed.

#### 2.3.1 MUNE examinations

Routine sensory and motor conduction studies were performed before MUNE testing to rule out neural variations. The MUNE program in the electromyography instrument was used, with the stimulating electrode placed between the flexor carpi radialis and palmaris longus tendons at the wrist. The recording electrode was placed on the belly of the abductor pollicis brevis muscle, and the reference electrode was positioned at the distal tendon of the abductor pollicis brevis. The ground electrode was placed between the recording and stimulating electrodes. After identifying the optimal stimulation and recording points, stimulation was performed using a handle electrode, and recordings were made using surface electrodes. The incremental method was employed, providing increasing intensity nerve stimulation from sub-threshold levels to obtain compound muscle action potentials (CMAP) in the abductor pollicis brevis. The mean amplitude of single motor-unit action potentials (S-MUAPs) was derived from the incrementally increased amplitudes. The MUNE was calculated as the ratio of the maximum CMAP amplitude to the mean S-MUAPs amplitude, with the computer program automatically computing the MUNE.

#### 2.3.2 F-wave examinations

The positions of the stimulating and recording electrodes were the same as in the MUNE examination. Supramaximal electrical stimulation was administered 20 times consecutively to record F-waves. The study used the mean latency and mean amplitude of F-waves. Repeater F-waves (17) were defined as F-waves with identical waveform, latency difference not exceeding 0.5 ms, and amplitude difference not exceeding 0.1 mV. Repeater F-waves were identified through visual inspection, requiring the superimposition of 20 traces to identify consistent shapes, with the entire waveform from start to return to baseline needing to be identical. Any interruptions or extra phases disqualified the waveform from being a repeater F-wave. A-waves, characterized by 4–8 unchanged waveforms with almost constant latency (within 1.5–4.0 ms), were excluded from this study. The occupancy rate of repeater F-waves (ORF) was calculated as the number of repeater F-waves divided by the total number of F-waves, expressed as a percentage. The occupancy rate of non-repeater F-waves (non-ORF) was derived by subtracting the ORF from 100%.

### 2.4 Statistical methods

Data were processed using SPSS 25.0 software. Measurement data conforming to a normal distribution were expressed as mean ± standard deviation (x ± s), and intergroup differences were analyzed using *t*-tests or one-way ANOVA. Measurement data not conforming to a normal distribution were expressed as median (lower quartile, upper quartile), with differences analyzed using the rank-sum test. Multiple comparisons were corrected using the Bonferroni method. Count data were presented as frequency and percentage, with intergroup comparisons made using the *χ*^2^ test. Pearson correlation analysis was used to explore correlations between indicators, and multiple linear regression and binary logistic regression were employed for multifactor regression analysis. ROC curve analysis was conducted to assess the predictive ability of various indicators for the improvement of FMA-UE 1 year after discharge. The significance level (*α*) was set at 0.05.

## 3 Results

### 3.1 Baseline characteristics

According to the inclusion and exclusion criteria, 16 patients were excluded due to a disease course exceeding 6 months. During the follow-up process, 41 patients were lost to follow-up, and 3 patients died. Ultimately, 130 stroke patients were included in the study (Table 1). All participants included in the study were confirmed right-hand dominant. The average age of the patients was 60.62 ± 13.48 years. Among them, 95 were male (73%) and 35 were female (27%); 109 had cerebral infarction (84%) and 21 had cerebral hemorrhage (16%). The disease duration was less than 1 month in 77 patients (59%), 1–3 months in 36 patients (28%), and 3–6 months in 17 patients (13%). There were 63 patients (48%) with left-sided hemiplegia and 67 patients (52%) with right-sided hemiplegia. Muscle tone was decreased in 21 patients (16%), normal in 59 patients (45%), and increased in 50 patients (39%).

**Table 1:** Baseline characteristics of the patients (*n* = 130).

Variable	Values
Age (years)	
Mean ± SD	60.62 ± 13.48
Gender, *n*	
Male/Female	95/35
Stroke, *n*	
Ischemic/Hemorrhagic	109/21
Disease duration (month), *n*	
Average (range)	0.85 (0.43, 1.63)
<1	77
1–3	36
3–6	17
Hemiplegic UE, *n*	
Left/right	63/67
MAS, *n*	
Decreased	21
0	59
1	25
1+	16
2	8
3	1

### 3.2 Comparison of upper extremity neuroelectrophysiological assessment

**Figure 1:**
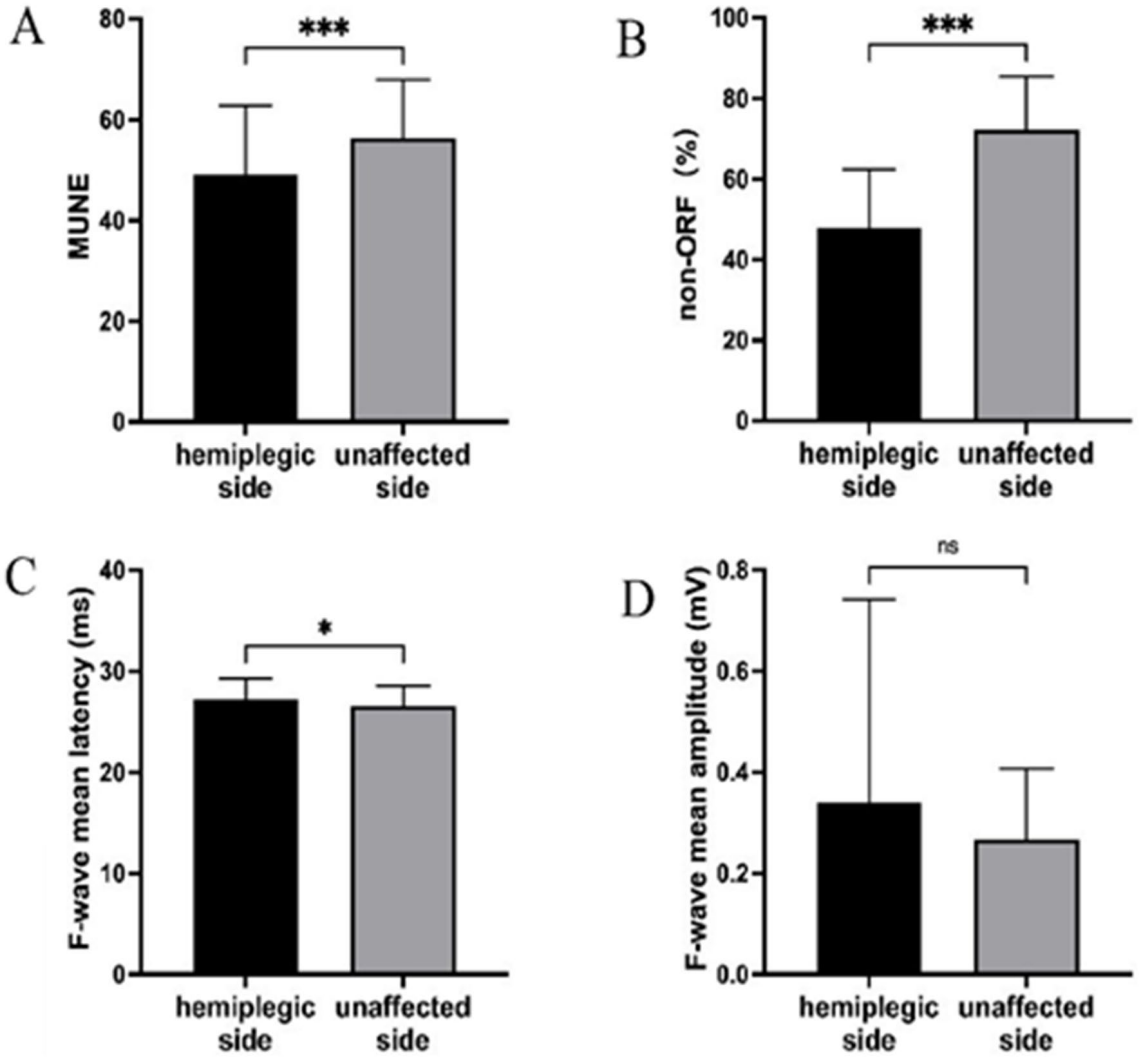
The results of the UE neuroelectrophysiological assessment. **(A)** MUNE; **(B)** non-ORF; **(C)** F-wave mean latency; **(D)** F-wave mean amplitude. *** Indicates the hemiplegic side is significant lower (*p* < 0.001) than the unaffected side. * Indicates the hemiplegic side is higher (*p* < 0.05) than the unaffected side. ns, Indicates the hemiplegic side is not significant the unaffected side.

We first analyzed the changes in neuroelectrophysiological assessment between the hemiplegic and unaffected sides (Table 2 and Figure 1). The results showed significant statistical differences in MUNE and non-ORF between the hemiplegic and unaffected sides (*p* < 0.001). The difference in the mean latency of F-waves was also statistically significant (*p* < 0.05). However, there was no statistically significant difference in the mean amplitude of F-waves between the hemiplegic and unaffected sides (*p* > 0.05).

**Table 2:** Comparison of the results of the UE neuroelectrophysiological assessment.

	Hemiplegic side	Unaffected side	Statistic	*p* value
MUNE	49.12 ± 13.71	56.34 ± 11.49	*t* = 4.607	<0.001
Non-ORF (%)	47.92 ± 14.56	72.21 ± 13.21	*t* = 14.086	<0.001
F-wave mean latency (ms)	27.18 ± 2.10	26.57 ± 2.01	*t* = −3.337	0.016
F-wave mean amplitude (mV)	0.23 (0.14, 0.37)	0.24 (0.16, 0.35)	*z* = −0.224	0.822

### 3.3 Correlation between admission FMA-UE and neuroelectrophysiological assessment

**Figure 2:**
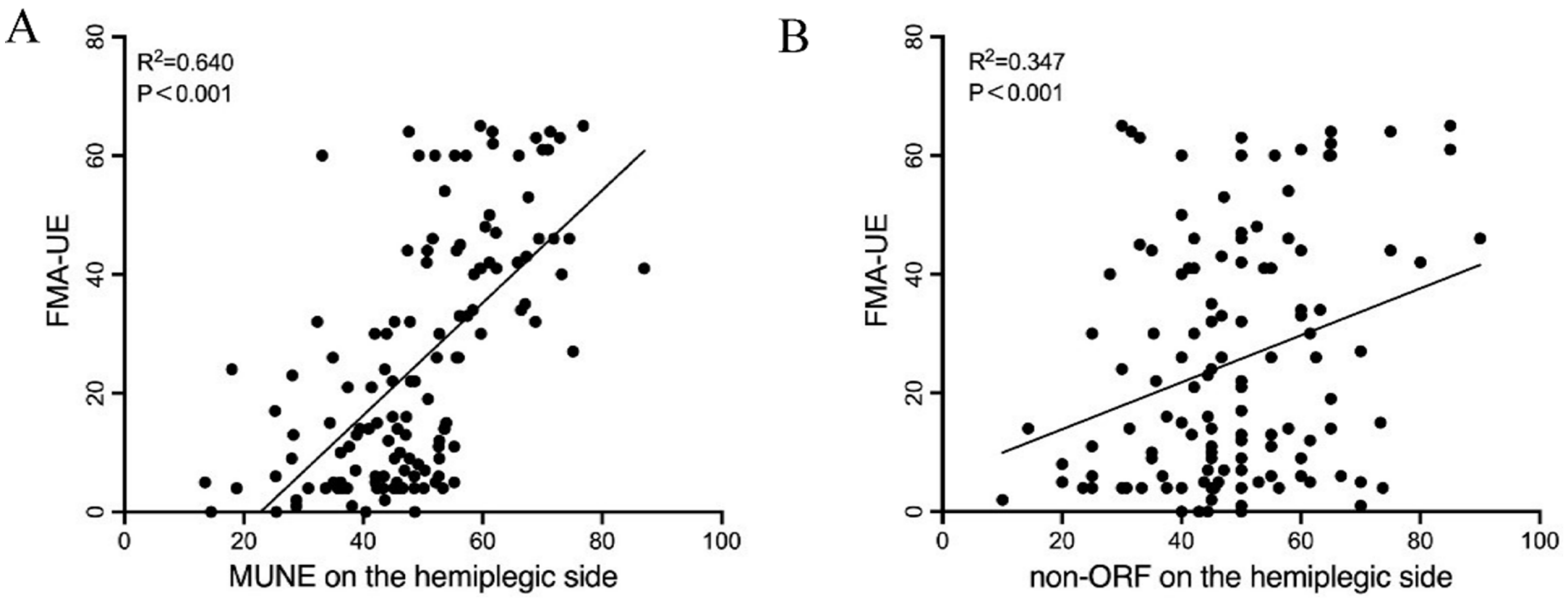
Pearson correlation analysis between FMA-UE and UE neuroelectrophysiological assessment in patients. **(A)** MUNE on the hemiplegic side; **(B)** non-ORF on the hemiplegic side.

Pearson correlation analysis showed that the FMA-UE at admission was significantly correlated with the hemiplegic side’s MUNE (*r* = 0.640, *p* < 0.001) and the hemiplegic side’s non-ORF (*r* = 0.347, *p* < 0.001) (Table 3 and Figure 2). There was no significant correlation between FMA-UE and the unaffected side’s MUNE (*r* = 0.097, *p* = 0.270), the hemiplegic side’s mean latency of F-waves (*r* = 0.156, *p* = 0.076), the unaffected side’s mean latency of F-waves (*r* = 0.211, *p* = 0.060), or the unaffected side’s non-ORF (*r* = 0.064, *p* = 0.473).

**Table 3:** Pearson correlation analysis between FMA-UE and UE neuroelectrophysiological assessment.

	MUNE		F-wave mean latency (ms)		Non-ORF (%)	
	Hemiplegic side	Unaffected side	Hemiplegic side	Unaffected side	Hemiplegic side	Unaffected side
*r*	0.640	0.097	0.156	0.211	0.347	0.064
*P*	<0.001	0.270	0.076	0.060	<0.001	0.473

### 3.4 Multiple linear regression analysis

Based on the correlation analysis results, multiple linear regression analysis was conducted with age, gender, disease duration, type of stroke, side of hemiplegia, MAS, hemiplegic side MUNE, and hemiplegic side non-ORF as independent variables, and FMA-UE at admission as the dependent variable. The results indicated that the hemiplegic side’s MUNE (*β* = 0.88, *p* < 0.001) and the hemiplegic side’s non-ORF (*β* = 0.275, *p* = 0.005) were influencing factors for FMA-UE (Table 4). This suggests that, after adjusting for confounding factors, the hemiplegic side’s MUNE and non-ORF significantly impact the FMA-UE at admission.

**Table 4:** Multiple linear regression analysis of influencing factors of FMA-UE in patients at admission.

Variant	Standard error	Β (95%CI)	*t* value	*P* value
Constant	9.771		−3.222	0.002
Age	0.108	−0.074 (−0.287, 0.139)	−0.689	0.492
Gender	3.161	0.982 (−5.277, 7.241)	0.311	0.757
Disease duration	1.266	2.164 (−0.343, 4.672)	1.709	0.09
Stroke type	3.701	2.405 (−4.923, 9.733)	0.65	0.517
Hemiplegic UE	2.793	0.312 (−5.217, 5.841)	0.112	0.911
MAS	1.413	−2.383 (−5.181, 0.414)	−1.687	0.094
MUNE on the hemiplegic side	0.103	0.88 (−0.676, 1.085)	8.518	<0.001
non-ORF on the hemiplegic side	0.097	0.275 (−0.084, 0.467)	2.844	0.005

SE, Standard error; *β*, Standardized coefficient; CI, Confidence interval.

### 3.5 FMA-UE at one-year follow-up

**Figure 3:**
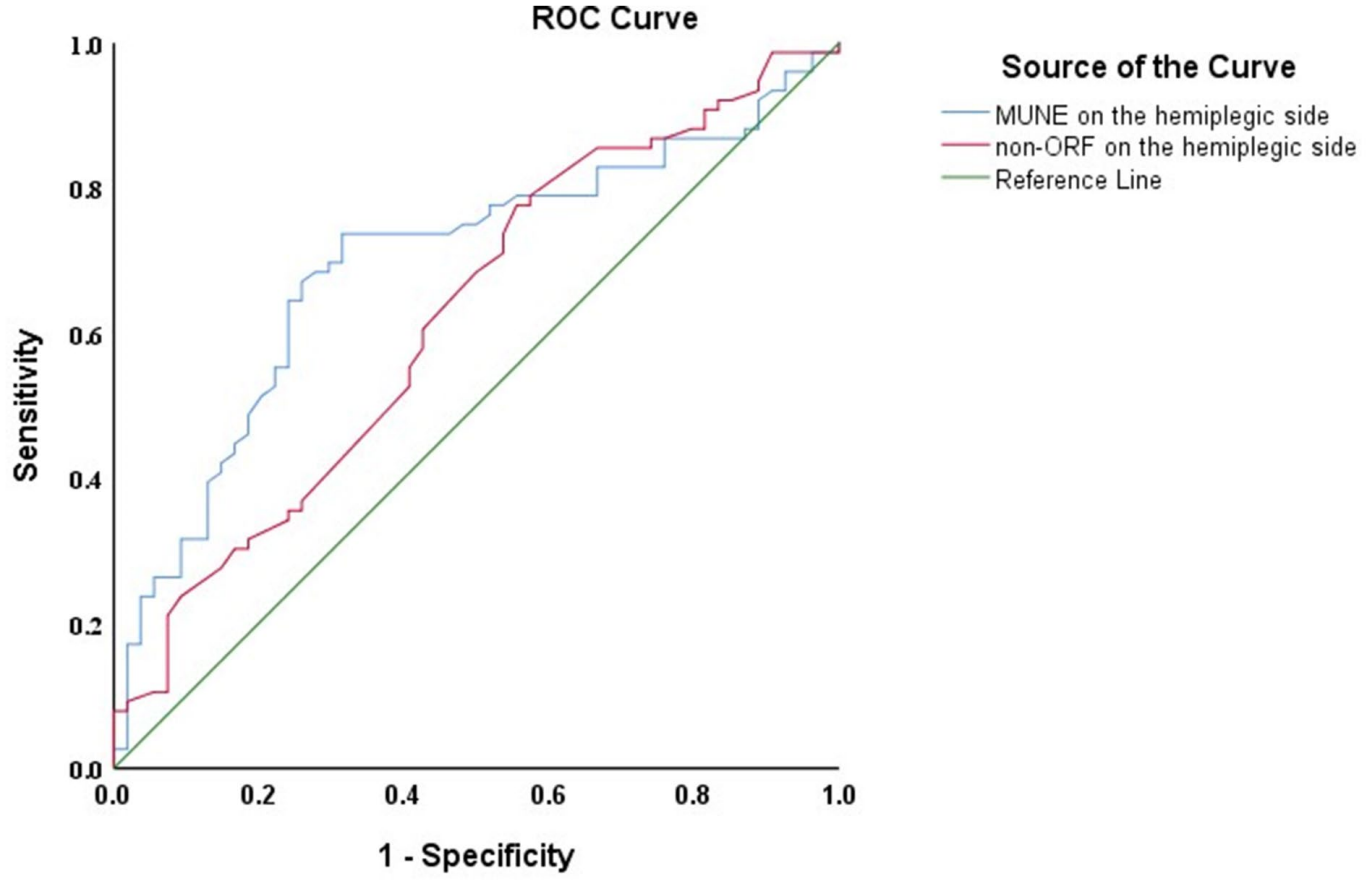
ROC curves of the hemiplegic side MUNE and non-ORF for predicting FMA—UE 1 year after discharge in patients.

Follow-up results showed that among the 130 patients, 76 exhibited significant improvement (ΔFMA-UE ≥ 6 points). ROC curve analysis was used to assess the predictive ability of the hemiplegic side’s MUNE and non-ORF for FMA-UE improvement 1 year after discharge. The results (Figure 3) indicated that both the hemiplegic side’s MUNE and non-ORF could predict the improvement in FMA-UE 1 year post-discharge. Additionally, the predictive ability of the hemiplegic side’s MUNE for FMA-UE improvement (AUC = 0.696, *p* < 0.001) was higher than that of the hemiplegic side’s non-ORF (AUC = 0.622, *p* = 0.018).

### 3.6 Binary logistic regression analysis

Binary logistic regression analysis was conducted on the variables to determine the improvement in FMA-UE 1 year after discharge. After adjusting for confounding factors such as age, the analysis showed that the hemiplegic side’s MUNE was a significant risk factor for FMA-UE improvement 1 year post-discharge (*p* < 0.05) (Table 5).

**Table 5:** Binary logistic regression analysis of FMA-UE improvement in patients one year after discharge.

Variant	Define	*b*	SE	OR (95%CI)	*p* value
Constant	–	−2.994	1.466	0.05	0.041
Age	<62 = 0, ≥ 62 = 1	−0.003	0.016	0.997 (0.967, 1.027)	0.822
Gender	Female = 0, Male = 1	0.527	0.445	1.695 (0.708, 4.055)	0.236
Disease duration	<0.87 = 0, ≥ 0.87 = 1	−0.059	0.185	0.943 (0.656, 1.355)	0.75
Stroke	Hemorrhagic = 0, Ischemic = 1	−0.024	0.538	0.977 (0.34, 2.805)	0.965
Hemiplegic UE	Left side = 0, right side = 1	0.515	0.406	1.674 (0.755, 3.71)	0.204
MAS	<1 = 0, ≥ 1 = 1	−0.278	0.204	0.757 (0.508, 1.128)	0.172
MUNE on the hemiplegic side	<48.6 = 0, ≥ 48.6 = 1	0.043	0.016	1.044 (1.012, 1.076)	0.006
Non-ORF on the hemiplegic side	<47.1 = 0, ≥ 47.1 = 1	0.023	0.014	1.023 (0.995, 1.053)	0.114

## 4 Discussion

Stroke is characterized by high incidence, high mortality, and high disability rates, making it a serious global healthcare issue (18). In recent years, with the advancements in neurophysiology and imaging, researchers have been focusing on how to use precise, quantitative, non-invasive methods to assess and predict the recovery of motor dysfunction after stroke (19). Neuroelectrophysiological techniques, as an extension of nervous system examinations, offer high sensitivity and detect changes before clinical signs appear. These techniques provide relatively objective indicators for estimating prognosis, evaluating treatment efficacy, and selecting treatment plans for stroke (20). This study explored the neuroelectrophysiological data of stroke patients, analyzing the functional status of motor neurons post-stroke and its association with upper extremity motor function. This helps in understanding the extent of upper extremity motor function at an early stage and implementing effective rehabilitation interventions promptly.

Currently, clinical assessment of motor neuron function relies heavily on functional scales, lacking quantitative, reproducible evaluation evidence that accurately reflects the functional status of motor neurons. The MUNE indicator used in this study quantitatively evaluates the total number of functional motor units in a muscle or muscle group innervated by a nerve from a macro perspective. MUNE values represent the number of functional motor units, which is crucial for predicting the prognosis of motor function recovery in patients (21, 22). The abductor pollicis brevis muscle is often chosen for MUNE due to its responsiveness to electrical stimulation and good reproducibility. Our findings align with those of Hara et al. (12), who found a reduction in the number of motor units in the hemiplegic abductor pollicis brevis muscle post-stroke, confirming the difference in motor unit numbers between the hemiplegic and unaffected sides. This reduction in motor units post-stroke may result from transsynaptic degeneration of lower motor neurons caused by upper motor neuron damage. Another study by Hara et al. (23) indicated that the reduction in motor units on the hemiplegic side might occur in the second week after upper motor neuron lesions and is closely related to the severity of motor dysfunction. Patients with severe hemiplegia experience a greater reduction, which persists up to 1 year post-stroke. This reduction in functional motor units may affect the patient’s ability to generate muscle force and control fine movements, thereby impacting the motor function of the upper limb. Future research should focus on finding methods to prevent the reduction of functional motor units due to transsynaptic degeneration of lower motor neurons in the acute phase of stroke, which could enhance functional recovery and improve daily living abilities.

Lower motor neurons mainly include anterior horn motor neurons of the spinal cord and their subsequent nerve roots and peripheral nerves (24). After losing higher central control, anterior horn motor neurons can transition from early inhibition to excitation, clinically presenting as a progression from flaccid paralysis with decreased muscle tone to increased muscle tone, spasticity, and eventual recovery of voluntary motor control (25). Studies have shown that the functional status of anterior horn motor neurons post-stroke reflects the physiological basis of muscle function in the hemiplegic limbs. Terao et al. (26) demonstrated that the loss of trophic support from upper motor neurons in stroke patients can alter the functional status of cells in the hemiplegic anterior horn of the spinal cord without losing the cells themselves. Qiu et al. (6) found no significant difference in the number of anterior horn cells between the hemiplegic and unaffected sides in stroke patients compared to normal subjects, but the cross-sectional area of the hemiplegic anterior horn cells was significantly reduced. Therefore, anterior horn motor neurons might be in a state of functional inhibition. Reactivating these inhibited anterior horn motor neurons could potentially aid in the functional improvement of stroke patients (27).

Studies have shown that F-waves can reflect the excitability of anterior horn motor neurons and assess the integrity of motor neurons and motor pathways (28). Measuring the mean latency of F-waves provides a sensitive and reliable method for examining the conduction properties of the proximal segment of motor axons. In this study, the mean latency of F-waves on the hemiplegic side was significantly different from that on the unaffected side, with the latency being prolonged on the hemiplegic side. This indicates that the conduction of functionally active motor neurons may be impaired after a stroke, consistent with previous findings (29, 30). Post-stroke, synaptic degeneration of upper motor neurons and increased excitability of anterior horn motor cells result in prolonged mean latency of F-waves, demonstrating the high sensitivity of F-waves to spinal excitability changes. This makes F-waves a probe for excitability changes in anterior horn cells, serving as an objective indicator of anterior horn motor cell excitability (31, 32).

Additionally, our research team conducted a statistical analysis of repeater F-waves in stroke patients. Repeater F-waves are generated by repetitive impulses from a single motor neuron. Their presence has been confirmed in conditions like carpal tunnel syndrome (33), motor neuron diseases, cervical spondylosis (34), lumbosacral radiculopathy (35), and poliomyelitis (36). Repeater F-waves can enhance the sensitivity of diagnosing certain neuromuscular disorders. Despite their recognized importance, quantitative evaluation of repeater F-waves in stroke patients is uncommon. This study found a decrease in non-ORF on the hemiplegic side, indicating an increase in the proportion of repeater F-waves. This suggests that fewer motor neurons generate F-waves, possibly due to the central release of functionally active neurons exhibiting enhanced repetitive impulse generation (37, 38). As the number of motor neurons decreases, the proportion of F-waves with different shapes diminishes, while repeater F-waves from single motor neurons increase. Our findings indicate a reduction in the number of functional motor neurons on the hemiplegic side post-stroke, with the remaining neurons showing abnormally increased excitability, further validating the MUNE results. This increased excitability may represent a compensatory mechanism for the reduced descending motor input, suggesting that we can enhance motor function plasticity by modulating motor neuron excitability. Through changes in neurophysiological indicators such as MUNE and F-waves, we can observe alterations in both structural (loss of motor units) and functional (changes in spinal excitability) adaptations following stroke.

The correlation analysis between neuroelectrophysiological assessment and motor function showed a significant association between FMA-UE and the hemiplegic side’s MUNE and non-ORF. This indicates that a higher abundance of motor neurons generating impulses correlates with better motor function on the hemiplegic side. Multiple linear regression analysis revealed that, after adjusting for confounding factors, the hemiplegic side’s MUNE and non-ORF were significant influencing factors for FMA-UE at admission. ROC curve analysis suggested that the hemiplegic side’s MUNE and non-ORF have good predictive value for upper extremity motor function, with MUNE having a superior predictive ability compared to non-ORF.

One year post-discharge, follow-up analysis showed that the hemiplegic side’s MUNE remained a significant factor for the improvement in upper extremity motor function after excluding confounding factors like age. This indicates that MUNE is a good predictor of upper extremity motor function and is associated with motor function prognosis to some extent. Based on the results of this study, future research directions could explore neuroprotective strategies to mitigate transsynaptic degeneration of lower motor neurons in patients during the acute phase. Research could also focus on neuromodulation techniques to reactivate functionally inhibited anterior horn motor neurons in the spinal cord. Additionally, utilizing a multimodal assessment system that integrates neurophysiological evaluations and neuroimaging techniques could provide a more comprehensive understanding of structural and functional changes in both central and peripheral motor systems following stroke. This approach may lead to the development of more targeted and effective therapeutic interventions. However, this study has several limitations. First, it lacks electrophysiological and ultrasound evaluations of other peripheral nerves in the lower and upper extremities, which could provide additional information about the entire peripheral nervous system. Simultaneously, we did not consider the impact of disease severity, such as lesion size, location, and whether the patient had undergone cranial surgery, or other potential confounding factors on neurophysiological assessments. Additionally, comorbidities such as hypertension and coronary heart disease may also influence neurophysiological assessments and the recovery of upper limb motor function. Second, we did not perform needle electromyography, which could show denervation activity or evaluate motor unit potentials. Finally, more stroke patients and longer follow-up periods are needed to further investigate the relationship between neuroelectrophysiological assessment and upper extremity motor function.

In summary, neuroelectrophysiological assessment such as MUNE and F-waves of the median nerve are associated with FMA-UE, reflecting upper extremity motor function in stroke patients. These indicators have good predictive value for upper extremity motor function and are associated with motor function prognosis to some extent. The results suggest that MUNE and F-waves have potential predictive value for early assessment of motor function in stroke patients and support further related neuroelectrophysiological research.

## Data Availability

The raw data supporting the conclusions of this article will be made available by the authors, without undue reservation.
